# Acute Respiratory Failure after Administration of Hydrogen Peroxide as an Emetic in a Cat

**DOI:** 10.1155/2019/7242631

**Published:** 2019-09-25

**Authors:** Leona Rauserova-Lexmaulova, Carlos Agudelo, Barbara Prokesova

**Affiliations:** Small Animal Clinic, Faculty of Veterinary Medicine, University of Veterinary and Pharmaceutical Sciences, Palackeho tr. 1946, Brno 612 42, Czech Republic

## Abstract

**Objective and Case Summary:**

This case report describes a 5-year-old domestic short-haired cat that was orally administered with 4 mL of 3% hydrogen peroxide by the owner after suspecting ingestion of a foreign body by the cat. Shortly after the administration, the cat developed severe respiratory distress. Thoracic radiography showed an interstitial-to-alveolar pulmonary pattern, while echocardiography and heart injury markers ruled out a cardiac origin. Intensive management with oxygen, diuretics, bronchodilators, and sedation resulted in survival of the cat without further respiratory complications.

**New and Unique Information Provided:**

To the best of our knowledge, this is the first report of a lung injury and acute respiratory failure after administration of hydrogen peroxide in a cat with successful management.

## 1. Introduction

Hydrogen peroxide (H_2_O_2_) is a liquid chemical agent found in industrial and household products and medical preparations. It is commonly used in medicine as a cleaning agent and disinfectant. The effects of H_2_O_2 _depend on the release of nascent oxygen and effervescence on contact with the enzyme catalase on wound surfaces and mucous membranes [1, 2].

H_2_O_2_ is used as a common agent to induce emesis in dogs during first aid, but its use in cats is not recommended [2–4]. Solutions with an H_2_O_2_ concentration higher than 3% should not be orally administered because of the potential adverse effects, such as laryngospasm, foam formation in the mouth with a risk of aspiration, severe mucosal damage, repeated vomiting, hematemesis, necroulcerative gastritis (especially in cats), oxygen embolism, and systemic intoxication [3–7]. H_2_O_2_ inhalation may cause cough and transient dyspnea because of severe irritation and inflammation of the mucous membranes. Shock, coma, convulsions, and pulmonary edema may occur up to 24–72 h after exposure [1, 6].

This case report describes the successful management of respiratory distress in a cat following H_2_O_2_ administration by the owner. To the best of our knowledge, this event has not been previously described.

## 2. Case Presentation

A 5-year-old 3.6-kg castrated male domestic short-haired cat with no history of illnesses was referred to the University of Veterinary and Pharmaceutical Sciences Brno, Czech Republic because of lethargy, anorexia, and progression of labored respiration for one week. Seven days before the presentation, the cat had eaten a piece of string, and the owner had administered 4 mL of 3% H_2_O_2 _(in two doses, administered at a 15-min interval) via a syringe to induce vomiting. The owner reported that the cat did not vomit but made abnormal sounds (whistles) and gasped after the second dose of H_2_O_2_. A veterinary assessment was performed after 1 h by a private veterinarian. Clinical examination revealed salivation and an increased respiratory rate (RR) of 40 breaths/min (bpm), with no signs of an increased respiratory effort. The heart rate, body temperature, and mucous membranes were normal. The lung and heart auscultation and abdominal palpation were unremarkable, so no treatment was performed. In the evening, the condition of the cat worsened, and the owner returned to the veterinary clinic. RR was 60 bpm, with shallow respiration, cyanosis, wheezing, and bilateral crackles on chest auscultation. The body temperature was within the physiological range (37.8°C). The cat was hospitalized and stabilized using oxygen, cage rest, analgesia (10 mg/kg of IV metamizole every 12 h for the first 48 h), and antibiotics (30 mg/kg of IM amoxicillin clavulanate SID). Thoracic radiography after 12 h was unremarkable.

Endoscopy was performed on the fourth day. The mucous membrane was normal in the upper airway but irritated in the esophagus and stomach. No foreign bodies were detected. The cat presented with cardiovascular instability during anesthesia; therefore, bronchoalveolar lavage (BAL) was not performed.

Antibiotic treatment (30 mg/kg of IM amoxicillin clavulanate SID) and fluid therapy were continued, and dexamethasone (1 mg/kg IV BID, which was reduced to 0.25 mg/kg IV BID) and aminophylline (5 mg/kg IV BID) were added on the fourth day. After six days of therapy without improvement, the cat was referred to the Small Animal Clinic at University of Veterinary and Pharmaceutical Sciences.

**Figure 1:**
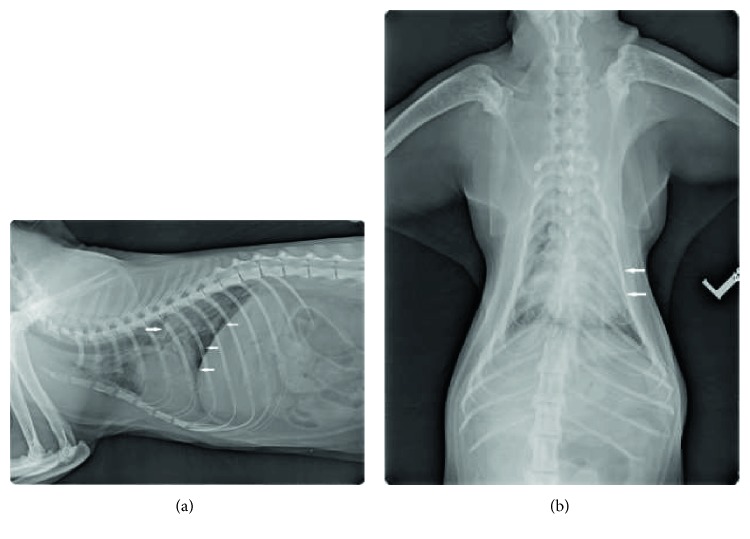
(a) Laterolateral right radiographic view: there is a mild right ventricular enlargement. Caudal lung field show/exhibit interstitial-to-alveolar patterns with air bronchograms (arrows). (b) Dorsoventral view. An alveolar lung pattern is apparent/obvious especially in the left cranial and caudal lung lobes (arrows).

Clinical evaluation at the time of admission revealed severe dyspnea, tachypnea (RR: 80 bpm), and cyanosis, bilateral crackles, normal heart sounds, and normal peripheral pulses. Abdominal palpation was unremarkable. For the stress response, intramuscular sedation (0.4 mg/kg of butorphanol, 0.1 mg/kg of midazolam, and 2 mg/kg of ketamine) and oxygen administration were performed before handling the cat. An intravenous catheter was placed, and blood samples (complete blood count and biochemistry) were collected. The results revealed thrombocytopenia (105 × 10^9^/L; reference range: 300–600 × 10^9^/L), hyperglycemia (12.9 mmol/L; reference range: 3.1–6.9 mmol/L), and mild hypokalemia (3.4 mmol/L; reference range 3.5–5 mmol/L). General anesthesia was induced and maintained with propofol (2 mg/kg IV to effect), and an intranasal catheter for oxygen administration was placed in the right nostril. Regarding the origin of dyspnea, thoracic radiography showed mild enlargement of the cardiac silhouette and alveolar lung pattern in the left cranial and caudal lung lobes (Figure 1). As the cat was anorectic for a few days, a nasoesophageal feeding tube was placed.

The initial differential diagnosis included pulmonary edema (noncardiogenic or cardiogenic) and pneumonia. Based on the history, clinical signs, laboratory results, and thoracic radiography, H_2_O_2_ aspiration with consequent noncardiogenic lung edema/pneumonia was suspected [8–11].

The oxygen therapy was continued, and a fluid therapy (Hartmann's solution with 0.1 mmol/kg/h of potassium supplementation) was started. Because of the cardiogenic etiology possibility of edema, 1 mg/kg of IV furosemide bolus was administered and repeated after 30 min, as RR did not decrease. After 2 h, 2 mg/kg of IV furosemide bolus was administered, as RR decreased from 80 to 59 bpm, and for the next 16 h, it was maintained at a constant rate infusion (CRI; 0.5 mg/kg/h). Additional supportive therapy included fentanyl CRI (2 *µ*g/kg/h), famotidine (0.6 mg/kg IV SID), and aminophylline (10 mg/kg IV TID).

**Figure 2:**
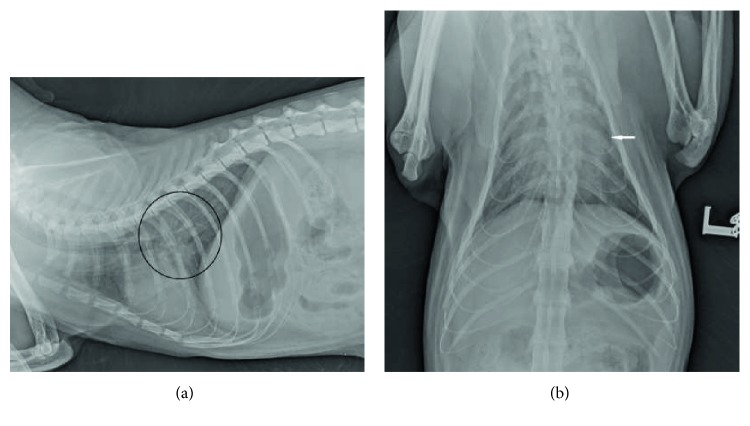
(a) Follow-up thoracic radiographs of the patient. Right lateral view: Caudal lung field showed noticeable resolution of described patterns (black circle). (b) Follow-up thoracic radiographs of the patient. Dorsoventral view. Decrease in radiopacity of the left caudal lung lobe (white arrow).

On day 2, the cat seemed to have improved, with a heart rate of 260 beats/min, RR of 46–56 bpm, pink mucous membranes, and subjectively decreased skin elasticity. Dyspnea improved, but after any manipulation, the respiration worsened; therefore, general anesthesia (0.1 mg/kg of midazolam, 2.0 mg/kg of ketamine, 1.5 mg/kg of propofol IV) was induced before examination. Auscultation revealed increased bronchovesicular sounds without crackles. Biochemistry, complete blood count analysis, electrocardiography (ECG), cardiac ultrasonography, and follow-up thoracic radiography were performed. Thoracic radiography showed smaller cardiac silhouette and improved pulmonary patterns (Figure 2). Hypokalemia persisted (2.7 mmol/L; reference range 3.5–5.0 mmol/L), and hypochloremia was also present (92.7 mmol/L; reference range: 111–125 mmol/L). ECG showed sinus tachycardia, and cardiac ultrasonography revealed hypertrophy of the interventricular septum and left ventricular posterior wall without left atrial enlargement. No evidence of diastolic dysfunction (including tissue Doppler imaging), valvular insufficiency, left or right ventricular outflow dynamic obstruction, or appendicular velocity abnormality was found [12, 13]. Coagulation profile and N-terminal pro-brain natriuretic peptide (NT-proBNP) on enzyme-linked immune sorbent assay were normal (42 pmol/L; reference range: <100 pmol/L), but cardiac troponin I was elevated (2.06 ng/mL; reference range <0.06 ng/mL) [14–19]. The absence of morphological and functional abnormalities in the heart suggested a noncardiogenic etiology.

On day 3, fentanyl administration was discontinued, and hypokalemia was corrected with the administration of 7.45% potassium chloride (0.4 mmol/kg/h in 2 h, followed by a dose of 0.1 mmol/kg/h as prophylaxis for hypokalemia recurrence). The flow rate of Hartmann's solution was slowly increased from one-fourth to one-half of the maintenance dose. Oxygen therapy and aminophylline and famotidine administration were continued. Furosemide CRI was reduced to 0.25 mg/kg/h for the next 24 h. Regular feeding via the nasoesophageal tube was continued, and CRI was slowly increased (up to 60% resting energy requirement).

On day 4, the cat was mentally stable and showed improved respiration (RR: 36–44 bpm); however, handling worsened the respiratory rate and dyspnea. The current therapy was continued unchanged, with the exception of furosemide, which was changed to intermittent bolus administration (1 mg/kg IV TID).

**Figure 3:**
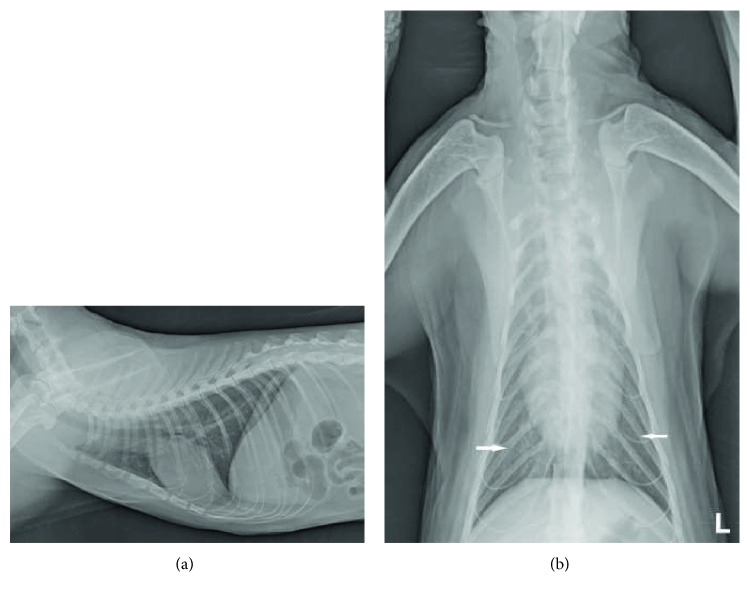
(a) Right lateral view. Lung lobes showed resolution of abnormal lung patterns. (b) Dorsoventral view. Presence of mild interstitial pattern in the caudal lung lobes (white arrows).

On day 5, RR was 32–36 bpm at rest, and the cat was clinically stable, with no signs of dyspnea or abnormal heart or lung sounds; only polyuria was noticed. Follow-up ECG findings were within the normal limits, and thoracic radiography showed only a mild interstitial pattern in the caudal lung lobes (Figure 3). The biochemical data revealed hyperglycemia (12.0 mmol/L; reference range: 4.4–7.7 mmol/L), an increased urea level (12.6 mmol/L, reference range: 5–11.3 mmol/L), hypomagnesemia (0.73 mmol/L; reference range: 1.8–2.4 mmol/L), hypophosphatemia (0.55 mmol/L; reference range: 0.9–28 mmol/L), and mild hypokalemia (3.4 mmol/L; reference range: 3.6–5.2 mmol/L) and hypochloremia (97.1 mmol/L; reference range: 114–123 mmol/L). Fluid therapy was changed to 5 mL/kg/h of PlasmaLyte (Ringer acetate gluconate) supplemented with 0.02 mmol/kg/h of potassium dihydrogen phosphate for 8 h. Oxygen supplementation, furosemide, and aminophylline were discontinued, but famotidine was continued. The cat was started on a normal diet and regularly fed with standard canned cat food.

On day 6, RR was 40 bpm, without signs of dyspnea, so the cat was sent home. Tramadol (1 mg/kg, tramadol drops) was recommended for pain management. Bed rest was mandatory. Over 2 years later, the cat was in good clinical condition, without signs of cardiac or respiratory diseases.

## 3. Discussion

To the best of our knowledge, this is the first report of acute respiratory distress induced by H_2_O_2 _administration and successfully managed in a cat. The use of 3% H_2_O_2 _administration as an emetic is not recommended in cats, and there are no reports describing acute respiratory distress after its administration [2–4]. Oral administration of 3% H_2_O_2_ solution in cats can result in necroulcerative gastritis as a sequela [3].

Serious lung injury with consequent noncardiogenic pulmonary edema/pneumonia from aspiration of chemical substances, resulting in conditions ranging from sensory irritation (including bronchospasm and cellular changes in the bronchioles and alveoli) to pulmonary diseases, has been described [7–10]. Inhalation of low-concentration H_2_O_2_ is a cause of upper airway irritation, inducing coughing, hoarseness, laryngospasm, and transient dyspnea and may peak within 30–90 min of intoxication, while pulmonary edema may occur within 24–72 h [7]. The owner of the cat reported abnormal sounds and dyspnea immediately after the second dose of H_2_O_2 _administration (due to laryngospasm), which had transiently disappeared one night but had worsened over the following days. H_2_O_2_ can also induce serious epithelial injury with consequent denudation, damage to the basement membrane, and endothelial cell dysfunction through a corrosive effect and lipid peroxidation (risk of reactive oxygen species generation) [1, 5]. Generation of a large amount of oxygen (1 mL of 3% H_2_O_2 _releases about 10 mL of free oxygen) results in embolization of the portal system, vena cava, right heart chambers, and arterial pulmonary system [1, 5–7, 9, 19–21] Leakage or interruption of the alveolar capillary membrane and fluid accumulation create a thicker diffusion barrier and worse mismatch between ventilation and perfusion, similar to cases of acute respiratory distress. This process is usually aseptic at first, as inflammation is a common pathophysiological response to injury, but secondary bacterial infection further compromises respiration and prolongs recovery. In this case, BAL was not performed during endoscopy. The condition became critical, so we suspected secondary pneumonia [8–10].

Respiratory distress has a wide range of differential diagnoses (heart disease, feline asthma, pneumonia, bleeding, thromboembolism, and inhalation of toxic substances) [10, 22]. Based on the history, clinical signs, and laboratory and radiographic findings, feline asthma, bleeding, and thromboembolism were less likely.

Fluid accumulation in pulmonary interstitial tissues and alveoli due to abnormal capillary membrane permeability can be detected on thoracic radiography and pulmonary ultrasonography [11, 12]. Localization of fluid accumulation is typical in the etiology in dogs but not in cats. Cardiac ultrasonography helped exclude cardiac diseases as the cause of pulmonary edema [11, 12, 23]. However, pulmonary diseases and dehydration affect the heart. This fact was consistent with the detection of wall thickening on ECG, suggestive of hypertrophic changes of the left and right heart chambers. However, the left atrial dimensions were normal, which indicated a noncardiac origin of edema and pseudo-hypertrophy [13]. and decreasing the furosemide dose, dimensions of the heart wall were normal [13]. Cardiac injury markers can be used to differentiate between cardiogenic and noncardiogenic pulmonary edema [14–19, 23, 24]. Natriuretic peptides are elevated in conditions that produce long-term stretching of the heart muscles (dilation of the cardiac chambers), such as primary heart diseases [18–19]. In our case, the values of NT-proBNP were within the normal ranges, and cardiac troponin I was slightly elevated [15–17]. Despite the specificity, cardiac troponins can also be elevated in cats with respiratory diseases caused by hypoxic myocardial injury [18]. This fact, in combination with normal values of the cardiac marker NT-proBNP and absence of signs of cardiac failure on ultrasound, suggested a non-cardiac pathology in this case [12, 15–18, 23, 24].

Both human and veterinary patients with noncardiogenic pulmonary edema may be treated with bed rest in the absence of severe clinical signs. However, in patients with severe edema and an interstitial-to-alveolar lung pattern on radiography, oxygen supplementation is mandatory, and mechanical ventilation could be additionally provided [22, 24].

Restoration of the normal lung fluid is an important component of the therapy in patients with pulmonary edema; however, administration of diuretics in veterinary cases of noncardiogenic pulmonary edema is controversial. Previous reports have recommended furosemide administration in both cardiogenic and noncardiogenic pulmonary edema [22–27]. Therefore, in the present case, a cardiac etiology could not be excluded at the time of admission, and furosemide was administered. Boluses were administered to rapidly induce diuresis and were continued with CRI to maintain a low dose of furosemide [22, 26]. Corticosteroids and bronchodilators are not beneficial in patients with both cardiogenic and noncardiogenic pulmonary edema [27].

Concurrent furosemide and aminophylline administration combined with anorexia predisposes patients to dehydration and acid–base and electrolyte imbalances [26]. The cat in this report showed hypokalemia, hypochloremia, hypomagnesemia, and hypophosphatemia, resulting from furosemide CRI, which induced diuresis, natriuresis, calciuresis, and kaliuresis. Secondary metabolic alkalosis induced an intracellular phosphorus shift with consequent hypophosphatemia. In addition, furosemide exhibits a weak carbonic anhydrase activity, which can explain its weak influence on phosphorus excretion [26].

Buffered solutions, such as Lactated Ringer's (Hartmann's) solution, are a possible choice, but goal-directed fluid therapy is necessary. In cases of cardiogenic pulmonary edema, IV fluid restriction as low as one-quarter of the maintenance rate or complete IV fluid restriction combined with furosemide CRI is recommended [28]. As cardiac diseases in this cat could not be excluded at the time of admission, one-quarter of the recommended IV fluid maintenance rate and furosemide were administered. Following improvement in ventilation, the fluid rate was slowly increased to manage dehydration. Since the applied fluid therapy was not efficient to maintain the normal potassium level, IV potassium supplementation was used to maintain normokalemia, as hypokalemia leads to muscle weakness, cardiac arrhythmias, and gastrointestinal tract hypomotility [29].

Irritation and inflammation of the mucous membrane in the pharynx, esophagus, and stomach, resulting from H_2_O_2_ ingestion and pain should be considered as a possible cause of inappetence. Some articles have reported the risk of hemorrhagic and necroulcerative hemorrhagic gastritis following 3% H_2_O_2_ administration as an emetic in cats; therefore, it is not recommended in this species [3, 6].

Nasoesophageal tube feeding in this case was preferred to force-feeding, which is stressful and potentially dangerous for patients with dyspnea. The calories were calculated based on the optimal body weight. The caloric intake was slowly increased for five days to prevent overfeeding and development of the refeeding syndrome [30].

The present case showed that H_2_O_2_ administration can be life-threatening and should be included in the differential diagnosis of patients with acute respiratory distress after the use of this agent as an emetic. It also showed that acute respiratory distress after H_2_O_2_ administration can be successfully managed with supportive care combined with transient diuretic administration. However, 3% H_2_O_2_ is not recommended for emesis induction in cats.
